# Evaluation of Endoglin (CD105) expression in pediatric rhabdomyosarcoma

**DOI:** 10.1186/s12885-017-3947-4

**Published:** 2018-01-05

**Authors:** Virginia Di Paolo, Ida Russo, Renata Boldrini, Lucilla Ravà, Marco Pezzullo, Maria Chiara Benedetti, Angela Galardi, Marta Colletti, Rossella Rota, Domenico Orlando, Alessandro Crocoli, Hector Peinado, Giuseppe Maria Milano, Angela Di Giannatale

**Affiliations:** 1 Department of Hematology/Oncology, Bambino Gesù Children’s Hospital, IRCCS, Piazza di Sant’Onofrio, 4, 00165 Rome, Italy; 2 Department of Laboratories - Pathology Unit, Bambino Gesù Children’s Hospital, IRCCS, Piazza di Sant’Onofrio, 4, 00165 Rome, Italy; 3 Clinical Epidemiology, Bambino Gesù Children’s Hospital, IRCCS, Viale Ferdinando Baldelli 41, 00146 Rome, Italy; 4 Core Facilities, Bambino Gesù Children’s Hospital, IRCCS, Viale San Paolo 15, 00146 Rome, Italy; 5 General Pediatric and Thoracic Surgery, Bambino Gesù Children’s Hospital, IRCCS, Piazza di Sant’Onofrio, 4, 00165 Rome, Italy; 6 Microenvironment and Metastasis Group, Molecular Oncology Program, Spanish National Cancer Research Centre (CNIO), C/ Melchor Fernández Almagro, 3, 28029 Madrid, Spain

**Keywords:** Rhabdomyosarcoma, Endoglin (CD105), CD105/CD31 ratio, Prognostic marker

## Abstract

**Background:**

The Intratumoral Microvessel Density (IMVD) is commonly used to quantify tumoral vascularization and is usually assessed by pan-endothelial markers, such as CD31. Endoglin (CD105) is a protein predominantly expressed in proliferating endothelium and the IMVD determined by this marker measures specifically the neovascularization. In this study, we investigated the CD105 expression in pediatric rhabdomyosarcoma and assessed the neovascularization by using the angiogenic ratio IMVD-CD105 to IMVD-CD31.

**Methods:**

Paraffin-embedded archival tumor specimens were selected from 65 pediatric patients affected by rhabdomyosarcoma. The expression levels of CD105, CD31 and Vascular Endothelial Growth Factor (VEGF) were investigated in 30 cases (18 embryonal and 12 alveolar) available for this study. The IMVD-CD105 to IMVD-CD31 expression ratio was correlated with clinical and pathologic features of these patients.

**Results:**

We found a specific expression of endoglin (CD105) in endothelial cells of all the rhabdomyosarcoma specimens analyzed. We observed a significant positive correlation between the IMVD individually measured by CD105 and CD31. The CD105/CD31 expression ratio was significantly higher in patients with lower survival and embryonal histology. Indeed, patients with a CD105/CD31 expression ratio < 1.3 had a significantly increased OS (88%, 95%CI, 60%–97%) compared to patients with higher values (40%, 95%CI, 12%–67%). We did not find any statistical correlation among VEGF and EFS, OS and CD105/CD31 expression ratio.

**Conclusion:**

CD105 is expressed on endothelial cells of rhabdomyosarcoma and represent a useful tool to quantify neovascularization in this tumor. If confirmed by further studies, these results will indicate that CD105 is a potential target for combined therapies in rhabdomyosarcoma.

**Electronic supplementary material:**

The online version of this article (10.1186/s12885-017-3947-4) contains supplementary material, which is available to authorized users.

## Background

Rhabdomyosarcoma (RMS) is the most common type of soft tissue sarcoma (STS) in children and young adults, accounting for up to 5% of all childhood cancers and for about 40% of pediatric STS [1]. Embryonal (ERMS) and alveolar (ARMS) RMS are the two major histologic subtypes. ARMS is associated with PAX3/7-FOXO1 gene fusions and with a poor prognosis, often being metastatic at diagnosis [2]. Although during the last three decades, multimodal treatment strategies have substantially improved the prognosis of localized RMS, for metastatic disease the prognosis remains dismal [3]. Therefore, new targets and tailored therapies directed against the metastatic process are needed for these patients. The formation of new blood vessels is a requirement for tumor growth and metastatic spread and many regulators of tumor angiogenesis have been identified in different types of cancer [4]. Studies on inhibitors of angiogenesis have shown antitumor activity in pediatric sarcoma models, including RMS, mostly in combination with other drugs [5–7], and several trials showed promising results for selected clinical indications [8–10]. The quantification of tumor vasculature is a useful indicator of angiogenesis, by helping patients stratification prior to anti-angiogenic therapy and monitoring patient response. One often-quantified aspect of tumor vasculature is the Intratumoral Microvessel Density (IMVD). IMVD is commonly used as a surrogate marker to quantify angiogenic activity and is usually assessed by pan-endothelial markers, such as CD34 and CD31 [10–13]. However, these markers are not tumor endothelial-specific, as they are also expressed on pre-existing/mature vasculature and on large vessels [14, 15]. Recent studies have shown that IMVD assessed by detection of Endoglin (CD105) is more specifically associated with tumor neovascularization [16–20] and represents a significant prognostic marker in several tumors [19–24]. CD105 is a transforming growth factor β (TGF-β) transmembrane co-receptor required for angiogenesis [25] and is highly expressed on the surface of actively proliferating microvascular endothelial cells, forming immature, highly permeable tumor neovessels [26]. In line with its supportive role in tumor neoangiogenesis, CD105 is up-regulated by hypoxia [27–29]. The expression of CD105 has been reported on the tumor vasculature of several sarcomas, including Kaposi sarcoma, angiosarcoma, leiomyosarcoma, chondrosarcoma and gastrointestinal stromal tumor and correlated with worse survival for some of these tumors [21, 30–33]. In this study, we aimed to investigate if CD105 was expressed in pediatric RMS and assess the neovascularization by using the angiogenic ratio IMVD-CD105 to IMVD-CD31. For this purpose, we evaluated the immunohistochemical expression of CD105, CD31 and VEGF in a retrospective series of pediatric patients with RMS. In order to define the proliferation fraction of the endothelium we compared the CD105 microvessels count with CD31 immunoexpression obtaining the CD105/CD31 expression ratio. In the cases where the CD105/CD31 expression ratio is higher, the angiogenesis is increased because CD105 marks the neoformed vessels [26] whereas CD31 is also expressed in mature vessels [18]. This ratio has been reported to have a prognostic value and be a potential predictor of response to anti-VEGF therapy [34–36].

## Methods

### Study population

Tumor tissue specimens from 65 patients with RMS who underwent surgical resection or biopsy of their primary tumor at the Bambino Gesù Children’s Hospital from 2005 to 2016 were retrospectively reviewed. Among these, we selected 30 appropriate paraffin embedded tissue blocks. The criteria for selecting the patients were based on the availability of an adequate tumor specimens obtained before any treatment and detailed clinical information. Patients’ clinical details, information on therapy and follow-up were collected retrospectively from the medical files. The median age at diagnosis was 48.5 months (range 1–199) with a sex ratio of 1. The most frequent primary site was head and neck (6 parameningeal and 2 non parameningeal patients respectively), followed by orbit (5 patients), pelvis (4 patients), genitourinary non-bladder or prostate (3 patients), extremity (2 patients), genitourinary bladder or prostate (1 patient), and other localizations (7 patients). This series include 18 patients with ERMS and 12 with ARMS. The study was approved according to local institutional guidelines.

### Patient variables analyzed

Patient- and tumor-related prognostic factors considered were: age at diagnosis (favorable if ≥ 12 or < 120 months and unfavorable if <12 or ≥ 120 months), primary tumor size (≤ 5 cm versus > 5 cm), tumor site favorable (orbit, genitourinary non bladder/prostate, head and neck non parameningeal) and unfavorable (parameningeal, extremities, genitourinary bladder-prostate and “other site”), histology (embryonal versus alveolar) and COG risk stratification [37].

### Immunohistochemistry methods

The tissues were fixed with 10% formalin and embedded in paraffin. Consecutive 2.5 μm-thick serial sections were cut, deparaffinised in xylene, rehydrated and washed using double distilled water. These sections were used for immunohistochemical staining for CD105, VEGF and CD31 and human tonsils were used as positive controls for CD105, CD31 and VEGF. For staining with VEGF and CD31, sections were pretreated with DAKO PT link (PT200) in low pH solution (cod. K8005 DAKO North America, CA) for antigen retrieval. As for CD105, sections were pretreated with Proteinase K (cod. S3020 DAKO North America, CA) for 10 min at room temperature. The immunostaining was done at 4 °C overnight using the following monoclonal mouse anti-human antibodies as primary antibody: anti-CD105 (clone SN6h, 1:10, DAKO North America, CA), anti-VEGF (MS-1467-P, 1:200, Thermo Fisher, Fremont, CA), anti-CD31 (IR610, Ready-to-Use, DAKO North America, CA). As the secondary antibody, we used En Vision Flex/HRP (cod. K8024, Ready-to-Use, DAKO North America, CA). The sections were then reacted in chromogen 3,3’-diaminobenzidine to detect the peroxidase activity, counterstained with hematoxylin and mounted.

### Measurement of IMVD

Hematoxylin-Eosin staining has been used by an experienced pathologist (RB) in order to select the area of the tumor, the necrotic areas were excluded. The sections were examined using a double-headed light microscope (Leica DM4 B) by two independent operators (RB and VDP), who were not aware of the clinical status of the patients. IMVD was assessed by immunostaining for either CD31 (IMVD-CD31) or CD105 (IMVD-CD105) according to the procedure described by Weidner et al., [11, 38]. The most vascularized area (hot-spots) was identified at low magnification (40X) and then three fields were counted at high magnification (20X). We considered as a countable single microvessel any endothelial cell or endothelial-cell cluster stained and clearly separated from the adjacent microvessels, tumor cells and other connective-tissue elements. The mean of the vessels in three fields was used as CD105 IMVD or CD31 IMVD. CD105 IMVD and CD31 IMVD have been evaluated in two different serial slides, within the same “hot spot” area. In order to define the proliferation fraction of the endothelium, we calculated the CD105/CD31 ratio dividing the IMVD of CD105 by the IMVD of CD31, as previously described [34–36]. Indeed, since CD31 is a pan-endothelial marker and CD105 is primarily expressed by proliferating endothelial cells, this ratio specifically measures the fraction of proliferating endothelial cells.

### Evaluation of VEGF

The VEGF expression was estimated according to the percentage of immunoreactive cells in a total of 1000 cells. The tumors were classified into 4 categories based on VEGF staining: negative (0), weak (1+), moderate (2+) and strong (3+). The percentage of positive cells was defined as sporadic (positive cells ≤ 1% and < 10%), focal (positive cells ≤ 10% and < 50%) or diffused (positive cells ≥ 50%). The immuno-histochemical scores were recorded as score 0 (no immunoreactivity), score 1 (1+ with sporadic or focal distribution), score 2 (1+ with diffused distribution or 2+ or 3+ with sporadic distribution), score 3 (2+ with focal or diffused distribution), score 4 (3+ with focal or diffused distribution) [39].

### Statistical analysis

Categorical data was represented as counts and proportions, and continuous data as mean and standard deviation or median and range. We analyzed the overall survival (OS) and event-free survival (EFS) defined as the time from diagnosis until the date of death and the date of disease relapse/progression, respectively. The follow-up period was calculated from the date of diagnosis until the last follow-up visit. Correlation between CD105 and CD31 IMVD was examined using the Spearman’s Rho. The ROC (Receiving operation curve) analysis was used in order to find an appropriate cut-off of CD105/CD31 ratio discriminating between death and survival, and event (disease relapse/progression) and non event in terms of sensibility and specificity.

Univariable analysis of time to event data (OS and EFS) was performed through the Kaplan Meier method, Log-rank test and Cox proportional hazard model. Relationships between the CD105/CD31 ratio and clinico-pathological data were assessed using univariable quantile regression analysis. *P* values less than 0.05 were considered to be statistically significant. Data was analyzed using the STATA software version 13.1.

## Results

### Clinico-pathological features of RMS patients

Patients’ characteristics are detailed in Table 1.

**Table 1:** Characteristics of the 30 patients with Rhabdomyosarcoma

Pt	Age (mos)	Gender	Histology	Primary site	Primary size (cm)	Metastasis	Nodes	IRS^a^ Group	COG Risk Groups	EpSSG Risk Groups	Status	CD105/CD31 ratio
1	83	M	ARMS	Extremity	> 5	No	N0	III	Intermediate	HR/G	NED	1.1611
2	65	F	ERMS	Pelvis	> 5	No	N1	III	Intermediate	HR/F	NED	0.6825
3	43	M	ERMS	Abdomen	> 5	No	N0	III	Intermediate	HR/E	NED	0.7466
4	82	F	ARMS	Extremity	> 5	No	N0	III	Intermediate	HR/G	NED	1.0460
5	55	M	ERMS	GU non BP	≤ 5	No	N0	I	Low	LR	NED	1.4292
6	41	M	ERMS	Orbit	> 5	Bone/BM	N0	IV	High	META	DOD	0.5133
7	28	F	ARMS	HN PM	≤ 5	No	N0	II	Intermediate	HR/G	NED	1.4225
8	13	M	ARMS	Orbit	≤ 5	No	N0	III	Intermediate	HR/G	NED	1.7949
9	11	F	ERMS	HN PM	≤ 5	No	N1	III	Intermediate	HR/F	DOD	1.3006
10	37	F	ERMS	GU non BP	≤ 5	No	N0	III	Low	SR/C	NED	0.8942
11	116	F	ERMS	Pelvis	> 5	Lung	Nx	IV	High	META	DOD	1.0158
12	176	M	ARMS	Retroperitoneum	> 5	Lung	N1	IV	High	META	DOD	1.5517
13	48	F	ERMS	GU BP	> 5	No	N0	III	Intermediate	HR/E	NED	1.8627
14	49	F	ARMS	HN PM	> 5	No	N1	III	Intermediate	VERY HR	NED	1.2461
15	17	M	ARMS	HN non PM	≤ 5	No	N0	II	Intermediate	HR/G	NED	1.0354
16	1	M	ERMS	Pelvis	> 5	No	N0	II	Low	HR/E	NED	0.8868
17	1	F	ARMS	HN PM	≤ 5	Multiple^b^	N1	IV	High	META	DOD	1.5608
18	16	F	ARMS	Chest wall	≤ 5	No	N0	II	Intermediate	HR/G	NED	0.8651
19	24	M	ARMS	Orbit	≤ 5	No	N0	III	Intermediate	HR/G	DOD	2.0024
20	135	M	ARMS	Chest wall	> 5	No	N1	III	Intermediate	VERY HR	DOD	1.9190
21	26	F	ERMS	Biliary tracts	≤ 5	No	N0	III	Low	SR/D	NED	1.1058
22	97	M	ERMS	Orbit	> 5	No	N0	III	Low	SR/C	NED	1.0274
23	154	M	ERMS	Orbit	> 5	No	N0	II	Low	SR/C	AWD	0.9020
24	14	F	ERMS	Abdomen	> 5	No	N1	I	Low	HR/F	DOD	2.3542
25	23	F	ERMS	Pelvis	> 5	Lung/Bone	N1	IV	High	META	NED	0.7117
26	199	F	ERMS	HN PM	> 5	No	N0	III	Intermediate	HR/E	AWD	0.9333
27	176	M	ARMS	GU non BP	≤ 5	Bone	N1	IV	High	VERY HR	NED	0.5914
28	106	F	ERMS	HN PM	> 5	No	N1	III	Intermediate	HR/F	AWD	1.0217
29	146	M	ERMS	HN non PM	> 5	No	N1	III	Low	HR/F	AWD	0.7733
30	189	M	ERMS	Extremity	> 5	No	N0	III	Intermediate	HR/E	AWD	0.3281

(*Pt* patient, *mos* months, *M* male, *F* female, *ARMS* alveolar rhabdomyosarcoma, *ERMS* embryonal rhabdomyosarcoma, *GU non BP* genitourinary non-bladder or prostate, *HN non PM* head and neck non-parameningeal, *GU BP* genitourinary bladder or prostate, *HN PM* Head and neck parameningeal, *BM* bone-marrow, *N0* no clinical or pathological node involvement, *N1* clinical or pathological nodal involvement, *NX* No information on lymph node involvement, *HR* High Risk, *META* metastatic, *LR* Low Risk, *SR* Standard Risk, *NED* no evidence of disease, *DOD* died of disease, *AWD* alive with disease, a: post-surgical stage according to Intergroup Rhabdomyosarcoma Study (IRS) grouping system41; b: subcutaneous, liver, pancreas, lung, bone-marrow and paravertebral lesion at L2-L3 level; COG, Children’s Oncology Group)

**Fig. 1:**
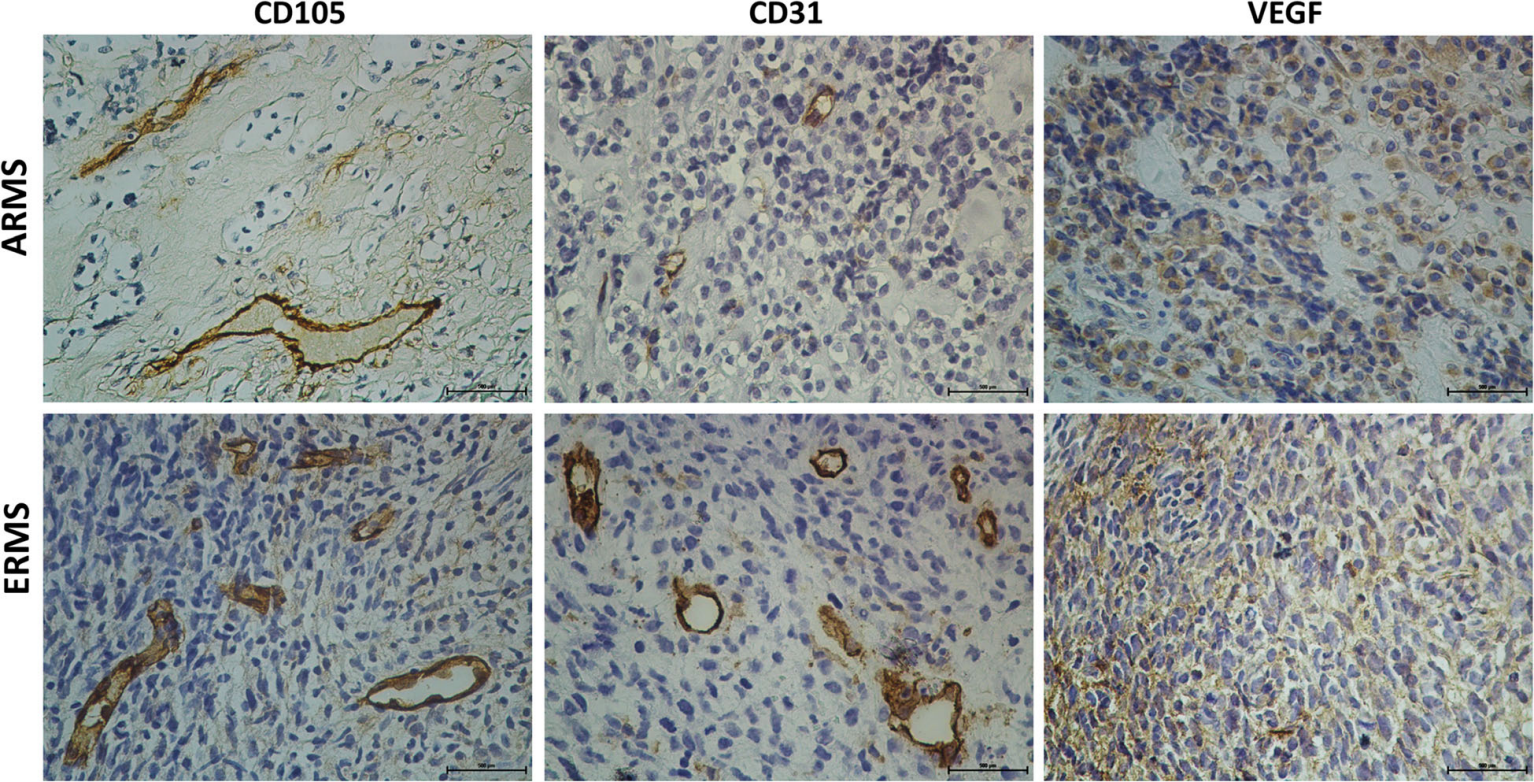
Representative immunostaining for CD105, CD31and VEGF of ARMS and ERMS. Magnification × 200

Patients were staged according to COG-STS risk stratification [37]. PAX3/PAX7-FKHR fusion gene transcripts were evaluated in 9 cases of 12 ARMS and 9 cases of 18 ERMS. PAX3-FKHR fusion gene was positive in 8 ARMS cases, and PAX7-FKHR in 1 ARMS case. None of PAX3/PAX7-FKHR fusion gene was detected in the ERMS examined. The median follow-up of patients was 5 years (range 0.28–11.12 years). Eight patients died of disease (median time from diagnosis 16.5 months, range 5–64). Patients #6, #17 and #24 presented a short follow-up since they died after 5, 7 and 10 months respectively due to rapid disease progression. The immunostaining was performed on pretreatment tumor biopsy specimens. The expression of CD31 and CD105 was localized in endothelial cells in all the specimens analyzed and not expressed by tumor cells. In the tumor CD105 was specifically associated with immature vessels which showed a stronger positivity compared to the large vessels (Additional file 1: Figure S1). VEGF expression was detected mainly in the cytoplasm of the tumor cells or endothelium (Fig. 1). Nineteen tumors (63.3%) showed a VEGF staining score of 1–2, while 11 (36.7%) showed a score of 3–4.

### The ratio of IMVD-CD105 to IMVD-CD31 in RMS primary samples

**Fig. 2:**
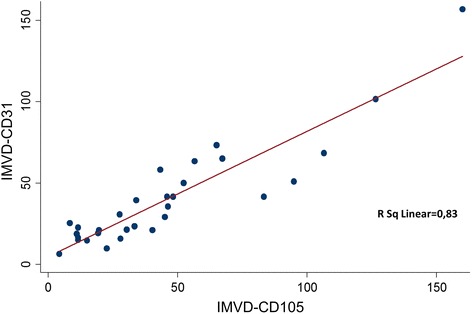
Correlation between CD105-IMVD and CD31-IMVD in rhabdomyosarcoma tumor samples (R2 = 0.83, *P* < 0.001)

**Fig. 3:**
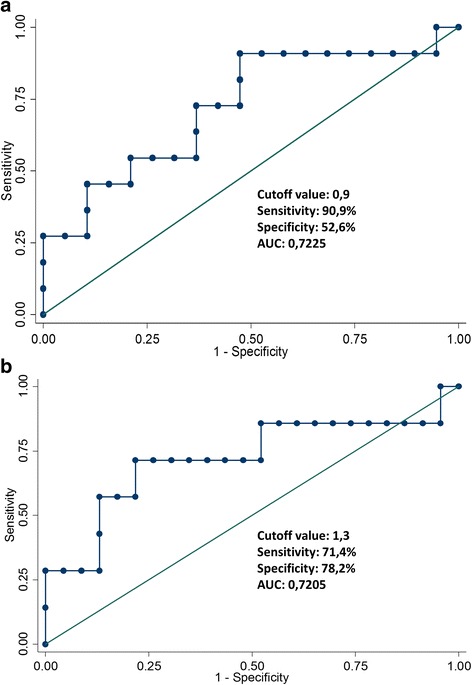
ROC analysis of CD31/CD105 ratio regarding Event-Free survival (**a**) and Overall survival (**b**)

Analysis of CD105 and CD31 expression demonstrated that the average of CD105-IMVD was not statistically, significantly higher than CD31-IMVD in RMS tissue (*P* = 0.122 Wilcoxon signed-rank test). We observed a statistically significant positive correlation between the IMVD individually measured by the two markers (Spearman’s rho = 0.86, *P* = 0.05), (Fig. 2). CD105/CD31 expression ratio in the tumor specimens ranged from 0.32% to 2.35%, with a median value of 1% and a mean of 1.15% (Table 1). The ROC curve analysis was used to determine the optimal cut-off of CD105/CD31 ratio (Fig. 3). EFS showed a cut-off point value of 0.9 with a 90.9% sensitivity and 52.6% specificity (Fig. 3a). OS had a cut-off point value of 1.3 with a 71.4% sensitivity and 78.2% specificity (Fig. 3b). Our analysis demonstrated that ten patients with a CD105/CD31 expression ratio equal or higher than 0.9 (50% of patients in this group) had relapse or disease progression. Only one patient (#6) with a ratio lower than 0.9 (10%) experienced disease progression, but had metastatic disease, which is per se a poor prognostic factor. These results suggest that the CD105/CD31 expression ratio in the primary tumor could be associated with disease aggressiveness.

### Correlation between prognostic factors and outcome in RMS patients

We then investigated the relationship amongst EFS or OS, selected prognostic clinico-pathological parameters (age at diagnosis, tumor size, primary site, histology, COG risk stratification) and the angiogenic CD105/CD31 ratio. As summarized in Table 2, the EFS and OS of patients with high risk RMS, according to COG stratification, resulted dismal, as it was previously reported [37].

**Table 2:** Univariable Cox proportional hazards regression for Event Free Survival and Overall Survival

Variables	EFS			OS		
	Hazard ratio	IC (95%)	P	Hazard ratio	IC (95%)	P
Age at diagnosis						
≥ 1 < 10 *(ref)*	–	–	–	–	–	–
< 1 ≥ 10	2.58	0.78;8.53	0.121	3.02	0.74; 12.25	0.121
Histology						
ARMS *(ref)*	–	–	–	–	–	–
ERMS	0.63	0.19; 2.06	0.443	0.86	0.21; 3.46	0.831
Tumor size						
≤ 5 cm *(ref)*	–	–	–	–	–	–
> 5 cm	1.66	0.44;6.28	0.453	2.17	0.43; 10.87	0.344
Primary site (location)						
Favorable *(ref)*	–	–	–	–	–	–
Unfavorable	1.11	0.32;3.80	0.867	1.14	0.27; 4.80	0.851
COG Group						
Low *(ref)*	–	–	–	–	–	–
Intermediate	2.93	0.35;24.41	0.319	1.13	011; 10.98	0.914
High	9.76	1.04;91.19	**0.046**	11.59	1.19; 112.25	**0.034**
VEGF score						
1–2 *(ref)*	–	–	–	–	–	–
3–4	0.54	0.14;2.06	0.373	0.46	0.93; 2.31	0.349
CD105/CD31 Ratio						
< 0.9 *(ref)*	–	–	–	–	–	–
≥ 0.9	5.31	0.75;46.25	0.090	–	–	**–**
CD105/CD31 Ratio						
< 1.3 (*ref)*	–	–	–	–	–	–
≥ 1.3	–	–	–	5.89	1.18;29.2	**0.030**

(*Ref* Reference, *IC* interval confidence, *COG* Children’s Oncology Group, *ERMS* embryonal rhabdomyosarcoma, *ARMS* alveolar rhabdomyosarcoma, *VEGF* Vascular Endothelial Growth Factor, *CD105* Endoglin). In boldface the values statistically significant

Furthermore, in the univariable Cox proportional hazard regression the CD105/CD31 expression ratio resulted to be related with decreased OS (*P* = 0.03) [38].

### Relationship of CD105/CD31 expression ratio with clinico-pathological characteristics and outcome

**Fig. 4:**
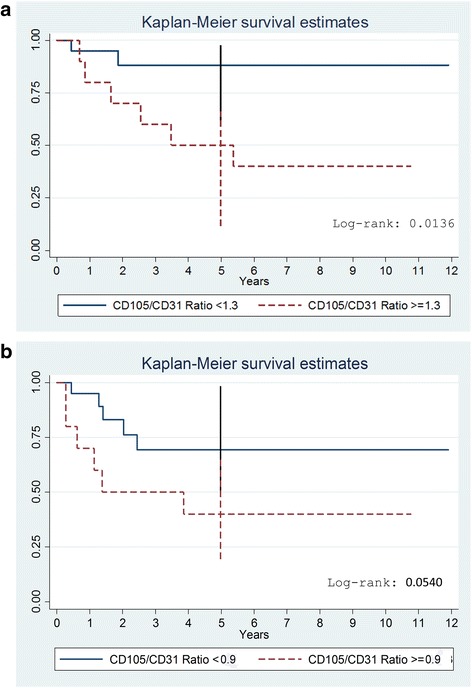
Kaplan-Meier curve for Event-Free survival (**a**) and Overall survival (**b**) according to CD105/CD31 ratio groups

Based on ROC curves cut-off values, Kaplan-Meier analysis showed that patients with a value of the CD105/CD31 expression ratio < 1.3 had a significantly increased OS (88%, 95%CI = 60%–97%) compared to patients with higher values (40%, 95%CI = 12%–67%; *P* = 0.013 by the log-rank test), (Fig. 4b). The estimated 5-year EFS was 91% (95%CI = 51%–98%) for patients with a CD105/CD31 ratio lower than 0.9 compared with 45% (95%CI = 22%–65%) for those with a ratio higher or equal to 0.9 (*P* = 0.054 by the log-rank test) (Fig. 4a). We further evaluated VEGF expression in order to correlate this marker, which is upregulated in RMS [39–43], with the neo-angiogenic ratio. Determinants of CD105/CD31 expression ratio were assessed by univariable quantile regression analysis (Table 3). This ratio was significantly associated with the patients’ survival (*P* = 0.016) and the embryonal histology (*P* = 0.019).

**Table 3:** Factors associated with CD105/CD31 expression ratio – univariable analysis

Variables	Number of patients (%)	Univariable Analysis		
		Coefficient	IC	*P* value
≥ 1 < 10 *(ref)*	21 (70)	–	–	–
< 1 ≥ 10	9 (30)	− 0.10	− 0.77;0.56	0.757
Histology				
ARMS *(ref)*	12 (40)	–	–	–
ERMS	18 (60)	− 0.49	− 0.89;-0.08	**0.019**
Tumor size				
≤ 5 cm *(ref)*	10 (33)	–	–	–
> 5 cm	20 (77)	− 0.28	− 0.83;0.27	0.313
Primary site (location)				
Favorable *(ref)*	12 (40)	–	–	–
Unfavorable	18 (60)	0.01	− 0.45;0.47	0.962
COG Risk Group				
Low *(ref)*	8 (27)	–	–	–
Intermediate	16 (53)	0.13	− 0.53;0.79	0.682
High	6 (20)	− 0.01	− 0.84;0.81	0.977
VEGF score				
1–2 *(ref)*	19 (63)	–	–	–
3–4	11 (37)	0.41	− 0.04;0.85	0.072
Status				
Alive *(ref)*	22 (73)	–	–	–
Dead	8 (27)	0.53	0.10; 0.95	**0.016**

(*Ref* Reference, *IC* interval confidence, *COG* Children’s Oncology Group, *ERMS* embryonal rhabdomyosarcoma, *ARMS* alveolar rhabdomyosarcoma, *VEGF* Vascular Endothelial Growth Factor, *CD105* Endoglin). In boldface the values statistically significant

## Discussion

Neo-angiogenesis has long been implicated in generating a microenvironment suitable for tumor growth and metastatic spread [44]. Several pro-angiogenic factors have been described and among them VEGF appears to play a central role in the activation of angiogenesis in various cancer [45, 46]. Several efforts have been made to develop therapies focused on the inhibition of the VEGF signaling pathway also in RMS [47, 48]. However these drugs led to transient responses and the complementary/dual inhibition of non-VEGF angiogenic pathways might represent a way to improve anti-angiogenic therapy. A phase I study using an anti-endoglin monoclonal antibody (TRC105) in combination with bevacizumab in adults with advanced cancers showed good tolerance and clinical activity in a VEGF inhibitor-refractory population [49]. A trial testing TRC105 in combination with pazopanib in patients with STS (≥12 years old) is currently ongoing [50]. In this context, methods which enable to quantify tumor angiogenesis are useful surrogate markers of angiogenic activity and response to therapy, and might help stratify patients with RMS for treatment. The IMVD is the most commonly used parameter to quantify intra-tumoral neovascularization and is measured by pan-endothelial markers, such as CD31. CD105 presents a higher specificity for new developing vessels and recent studies have shown that IMVD as determined by this marker has a higher prognostic impact than CD31 in several tumors [21–23]. In particular, IMVD ratio of CD105/CD31 expression, had been used to specifically assess neovascularization showing a prognostic value [34–36]. In the present study, we analyzed for the first time, the CD105 immunoexpression in pediatric RMS and quantified the presence of proliferating endothelial cells by using the CD105/CD31 expression ratio. CD105 was detected in small tumor capillary-like vessels, whereas CD31 presented a more diffused expression in endothelial cells. The significant positive correlation found between the IMVD measured by the two markers is coherent with the association between CD105 expression and other endothelial markers, such as CD31 and CD34, already described in other tumors [51, 52]. We also evaluated whether a correlation between this neoangiogenic ratio and clinic-pathological variables exists. Several prognostic factors, such as the age at diagnosis, primary tumor size, primary site, histology, post-surgical stage and presence or absence of distant metastases are currently used for risk-adapted treatment approaches in clinical trials of RMS patients [53]. Using these parameters, in the univariable survival analysis, we found that the advanced disease, classified according to the COG risk stratification, was a significant predictor of worse OS and EFS. In line with previously reported works, our study confirms that metastatic disease is the main prognostic factor in RMS [3]. The ROC curve for the OS indicated a cutoff point of 1.3, which was used to separate patients with good and poor prognosis. A value of the CD105/CD31 expression ratio < 1.3 was associated with a significantly better patients’ OS. These findings suggest that neovascularization could be an indicator of prognosis in patients with RMS and are supported by the correlation described between the CD105/CD31 expression ratio and aggressive phenotypes in other tumors [35]. Indeed, it has been already reported that IMVD quantified by CD105 correlate with poor survival in patients with breast carcinoma, non-small cell lung cancer and hepatocellular carcinoma [13, 54, 55]. Interestingly, when the histotype was specifically considered, we found that the ERMS correlated significantly with neo-angiogenesis. Kuda et al., previously described that IMVD, assessed by CD31, was higher in ERMS than ARMS [56]. We speculate that this association could be related to the different growth rate displayed by these two RMS histotypes. Indeed, although angiogenesis is a key process activated during cancer invasion and metastasis, highly aggressive histotypes are also able to support their growth through a process known as vasculogenic mimicry (VM) [57]. The generation of non-endothelialized vessel-like channels allows the perfusion of a variety of tumors, enabling them to aggressively proliferate and metastasize [58]. The VM channels are not lined by endothelial cells, but by tumor cells instead, and therefore are not stained by endothelial markers, including CD31 [59]. A higher incidence of VM has been described in tumors presenting necrosis, as well as in ARMS, and has been associated with poor prognosis [60, 61]. The faster growth of ARMS compared to ERMS may explain the different pattern of neovessels in the two variants. No statistically significant differences in CD105/CD31 expression ratio were encountered with respect to age, tumor size, primary tumor location, COG risk groups and VEGF. Despite VEGF overexpression has been reported to be associated with prognosis in RMS patients [42], data regarding the correlation amongst IMVD, VEGF expression and prognosis has shown conflicting results in several tumors including STS and RMS [39, 56, 62–64].

In conclusion, this small proof-of-concept study suggests that CD105 is expressed in endothelial cells of pediatric RMS and that CD105/CD31 expression ratio might be useful to measure the proportion of proliferating endothelial cells in this tumor. Despite the small cohort of patients studied, these data indicate that a high value of CD105/CD31 expression ratio could be related with a “pro-angiogenic” RMS subset of patients with low OS.

## Conclusions

If further studies confirm these results in larger cohorts of patients, CD105 may also represent a potential therapeutic target as part of combined therapy in RMS. In particular, an inter-institutional cooperative study would be advisable considering the low frequency of this tumor in the pediatric population. This type of large study could also be a tool to elucidate if the CD105/CD31 expression ratio may be useful for patient’s stratification and/or evaluate response to therapy.

## Additional file

Additional file 1: Figure S1. Immunostaining of CD105 for ERMS and ARMS. Magnification × 200. (PDF 266 kb)

## Abbreviations

AIEOP: Italian Association of Pediatric Hematology/Oncology
ARMS: Alveolar Rhabdomyosarcoma
CD105: Endoglin
COG: Children’s Oncology Group
EFS: Event-Free Survival
EpSSG: European Pediatric Soft Tissue Sarcoma Study Group
ERMS: Embryonal Rhabdomyosarcoma
IMVD: Intratumoral Microvessel Density
OS: Overall Survival
RMS: Rhabdomyosarcoma
STS: Soft Tissue Sarcoma
TGF-β: Transforming Growth Factor beta
VEGF: Vascular Endothelial Growth Factor
VM: Vasculogenic Mimicry
