# Supplementary material for: Evaluation of Endoglin (CD105) expression in pediatric rhabdomyosarcoma
Source: BMC Cancer. 2018 Jan 5;18:31. doi: 10.1186/s12885-017-3947-4 (PMC5755407; doi:10.1186/s12885-017-3947-4)
Supplement: Additional file 1: Figure S1. — Immunostaining of CD105 for ERMS and ARMS. Magnification × 200. (PDF 266 kb) [file 12885_2017_3947_MOESM1_ESM.pdf]

Patient#11 (ERMS)

Patient#12 (ARMS)

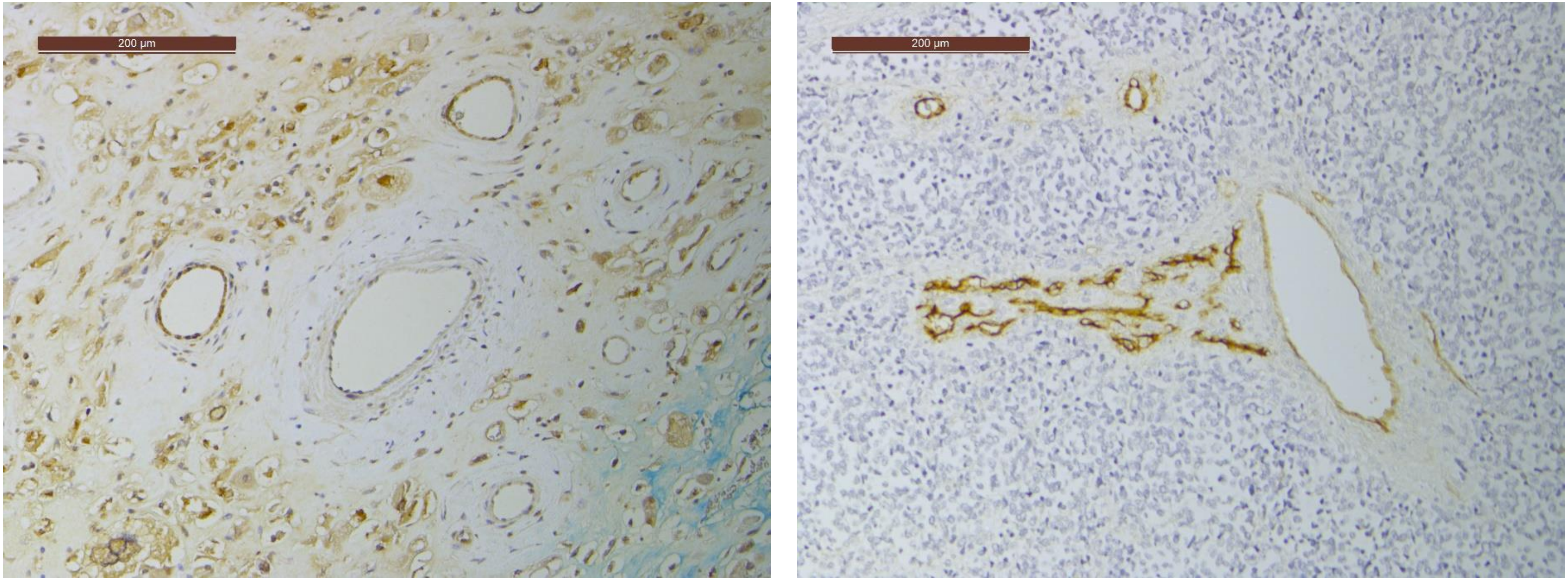

Supplemental Fig. 1: Immunostaining of CD105 for ERMS and ARMS. Magnification x 200.

The Pictures show that CD105 is specifically associated with new small developing vessels compared to large vessels in rhabdomyosarcoma.
